# Development and use of an index for measuring implementation of a weight management program in children in primary care clinics in Texas

**DOI:** 10.1186/s12875-018-0882-7

**Published:** 2018-12-05

**Authors:** Meliha Salahuddin, Sarah E. Barlow, Stephen J. Pont, Nancy F. Butte, Deanna M. Hoelscher

**Affiliations:** 1 Michael & Susan Dell Center for Healthy Living, University of Texas Health Science Center at Houston (UTHealth) School of Public Health in Austin, Austin, TX USA; 2 University of Texas Health Science Center at Tyler, Tyler, TX USA; 3 Population Health, Office of Health Affairs, University of Texas System, Austin, TX USA; 4 Texas Children’s Hospital, Baylor College of Medicine, Houston, TX USA; 5 UT Southwestern Medical Center, Dallas, TX USA; 6 Texas Center for the Prevention and Treatment of Childhood Obesity, Dell Children’s Medical Center, University of Texas at Austin Dell Medical School, Houston, TX USA; 7 USDA/ARS Children’s Nutrition Research Center; Department of Pediatrics, Baylor College of Medicine, Houston, TX USA

**Keywords:** Next steps, Weight management, Primary care clinics, Implementation index, Process evaluation, Children with overweight/obesity, TX CORD

## Abstract

**Background:**

The Texas Childhood Obesity Research Demonstration study was an integrated, systems-oriented intervention that incorporated primary and secondary obesity prevention approaches targeting multiple sectors, including primary care clinics, to address childhood obesity. The primary care clinic component included the American Academy of Pediatrics’ *Next Steps* weight management counseling materials that support brief healthy lifestyle-focused visits. The current study describes the methodology and assesses the implementation of the *Next Steps* program in the participating primary care clinics, as well as the association of implementation with enrollment of children with overweight and obesity in the secondary prevention intervention.

**Methods:**

The study used a serial cross-sectional study design to collect data from 11 primary care clinics in Houston (*n* = 5) and Austin (*n* = 6), Texas, in 2013–2014. Responses of primary care providers on 42 self-reported survey questions assessing acceptability, adoption, appropriateness, and feasibility of the program were utilized to create a mean standardized clinic implementation index score. Provider scores were aggregated to represent *Next Steps* implementation scores at the clinic level. A mixed effects logistic regression test was conducted to determine the association between program implementation and the enrollment of children in the secondary prevention.

**Results:**

Mean implementation index score was lower at Year 2 of implementation (2014) than Year 1 (2013) although the decrease was not significant [63.2% (12.2%) in 2013 vs. 55.3% (16.5%) in 2014]. There were no significant associations between levels of implementation of *Next Steps* and enrollment into TX CORD secondary prevention study.

**Conclusions:**

The development of an index using process evaluation measures can be used to assess the implementation and evaluation of provider-based obesity prevention tools in primary care clinics.

## Introduction

Over one-third of children in the United States have overweight or obesity, with more Hispanic, non-Hispanic black, and children from low socioeconomic conditions being affected than non-Hispanic white children [1, 2]. Primary care providers (PCP) are tasked with a periodic screening of children’s health and growth, and thus play a critical role in recognizing and addressing childhood obesity, a condition associated with heavy health and economic burdens [3–5]. However, at present, there is low active participation from PCPs in assessing or managing children with overweight or obesity in the primary care clinics [5], even though pediatricians can easily identify children with obesity [6]. Thus, it is essential to equip PCPs with effective prevention and treatment tools to address childhood obesity that they can easily implement.

Implementation research focuses on the processes and factors associated with successful integration of evidence-based programs within a particular setting, e.g., healthcare settings [7]. Many studies have implemented and assessed the effectiveness of different weight management programs in healthcare settings [8]; however, none, to the best of our knowledge, have developed and/or assessed the level of implementation of an intervention specific to weight management in healthcare settings using a composite score. Composite indicators are widely used in healthcare settings to measure and track provider performance as well as for external and internal benchmarking against other providers or institutions for quality improvement [9]. Developing a similar indicator or index for weight management interventions in primary healthcare settings will not only help to evaluate the fidelity of the intervention but also help to compare how the different primary care clinics perform.

The overall goal of this study was to describe the methodology and development of an index that can assess the implementation of the *Next Steps* program component of the Texas Childhood Research Demonstration (TX CORD) primary prevention study. The *Next Steps* program, which comprises weight management counseling materials [10], was pilot tested in the TX CORD study. The materials support brief counseling during busy office visits, with a goal of encouraging healthcare providers’ attention to obesity. Thus, it served as an engagement tool in study recruitment for the TX CORD primary prevention study and was also utilized in the comparison arm for the TX CORD secondary prevention study [11]. As a result, in our study, as a secondary objective, we examined the association of the implementation index of *Next Steps* with the enrollment of children with overweight and obesity in the TX CORD secondary prevention study.

## Method

### Study setting

#### The Texas childhood research demonstration (TX CORD) study

The TX CORD study was an integrated, systems-oriented model that incorporated primary and secondary obesity prevention approaches at multiple levels and sectors including primary care clinics in Houston and Austin, Texas, 2012–2014. The target population was low-income, underserved predominantly Hispanic and non-Hispanic black children aged 2–12 years [12, 13]. The primary prevention component was a quasi-experimental pre- and post-test community trial focused on improving healthy eating and physical activity in different settings, including primary care clinics.

#### Primary care clinic component of the TX CORD primary prevention study

Primary care clinics that served majority Medicaid, Children’s Health Insurance Program (CHIP), and uninsured population in the TX CORD catchment areas were invited to participate. The primary prevention component of the TX CORD study engaged five and seven primary care clinics from Houston and Austin, respectively. One Austin clinic was closed for remodeling during process data collection, and so this evaluation used data from the 11 participating primary care clinics.

All clinics received a set of coordinated counseling materials known as *Next Steps*, which were adapted for TX CORD and translated into Spanish. In Houston, an electronic health record (EHR) alert system was adapted from Taveras et al., (2014) and implemented to flag children with body mass index (BMI) ≥ 85th percentile, and to provide additional tools for the evaluation of comorbidities of obesity, obesity-related diagnostic codes, orders, and referrals [14]. Because Austin clinics were part of three different federally qualified health centers, with different EHR systems, the modifications were not implemented in the Austin EHRs. The providers received brief training in the use of the *Next Steps* materials, EHR changes (if applicable), and had a brief introduction to motivational interviewing techniques.

### Measures

#### Description of the next steps program

*Next Steps* is a set of coordinated weight management counseling materials for pediatric PCPs. These materials were created through a partnership with the National Initiative for Children’s Healthcare Quality, the Barbara Bush Children’s Hospital at Maine Medical Center, and the Maine Chapter of American Academy of Pediatrics (AAP) [10]. The materials included a menu of different healthy lifestyle themes, presented via poster and a desktop flip chart with a patient facing page and a provider-facing page with counseling tips. The TX CORD team also developed a patient home-based activity book, as a reinforcement tool for each theme that they gave to families at their discretion. Materials were available in both English and Spanish. Providers attended an in-person or webinar orientation to the material at the start of the project in 2012 and also received informal office visits from project staff two or three times per year between 2012 and early 2014, to ask questions and provide feedback.

#### Process evaluation survey

Providers, including those with MD, DO, nurse practitioner or physician assistant degrees, in the clinics, received a survey about the *Next Steps* material once in fall 2013 (Year 1 of implementation), approximately one year after the project started, and in fall 2014 (Year 2 of implementation). The *Next Steps* survey included data on provider characteristics, as well as different implementation measures [15], including acceptability, adoption, appropriateness, and feasibility of *Next Steps* program materials across the primary care clinics. These measures of implementation are in alignment with some of the measures for Diffusion of Innovation [16] and RE-AIM (Reach, Effectiveness, Adoption, Implementation, and Maintenance) [17, 18] implementation frameworks, which are further detailed below.

#### Provider characteristics

Data included provider gender, age, race/ethnicity, years of experience in the current position, medical field, working with pediatric patients after completion of medical training, and any prior training in the management of children with overweight and obesity.

#### Acceptability of using the next steps program materials

The acceptability construct included 3 items measuring providers’ ease of learning to use the materials and 6 items measuring their satisfaction with the materials. These were measured on a 5-point Likert scale from strongly disagree to disagree to neither agree nor disagree to agree to strongly agree. They were adapted from the *Next Steps* Guide: Feedback Survey [10]. This construct is supported by Roger’s “complexity” framework in the Diffusion of Innovation theory [15, 16].

#### Adoption of the next steps program materials

The adoption construct measured the frequency of use of the *Next Steps* program materials on a 5-point Likert scale ranging from less than 10% to 10–25% to 25–50% to 50–75% to 75–100%. The 5 items were developed by the TX CORD research team, and supported by the “adoption” framework from RE-AIM [17, 18] and Roger’s “trialability” framework from Diffusion of Innovation theories [15, 16].

#### Appropriateness of the next steps program materials

This construct measured providers’ perception of the effectiveness of the *Next Steps* program materials in treating children with overweight and obesity. These were measured on a 5-point Likert scale as well (strongly disagree to strongly agree). This construct also included whether the providers wanted any modification in any of the *Next Steps* healthy lifestyle themes encompassing three categories: Category A: Setting the foundation (*n* = 1 theme), Category B: Introduce concepts that are important to cover early for success (*n* = 5 themes), and Category C: Focus on other important concepts (*n* = 13 themes). Examples included understanding health, meaning of healthy food, home environment, themes around physical activity, body image, screen time, meal patterns and so on. The response options were on a 5-point Likert scale, ranging from “remove: theme not useful-remove from guide” to “reprioritize: like theme but it needs lower priority” to “edit: I like the theme, but content need revising” to “keep as is: I like theme as is” to “not applicable: I have not used this theme”. These were operationalized as binary variables, “keep as is” versus collapsing the rest of the groups. These items were adapted from the *Next Steps* Guide: Feedback Survey [10]. This construct is supported by Roger’s “compatibility” framework from Diffusion of Innovation theory [15, 16].

#### Feasibility of the next steps program materials

This construct included 4 items and measured whether the different *Next Steps* program components were made available to the providers by the TX CORD research team. They were developed by the TX CORD research team. This construct is supported by Roger’s “trialability” and “compatibility” framework from Diffusion of Innovation theory [15, 16].

### Study design and sample

The study utilized a serial cross-sectional study design. Process evaluation data were collected from 11 primary care clinics in Houston and Austin, Texas, respectively, in 2013 and 2014. These 11 primary care clinics were the participant recruitment source for the TX CORD secondary prevention study, which was a 12-month randomized controlled trial comparing intensive weight management programs among children with BMI ≥ 85th percentile and aged 2–12 years. Participant recruitment for the TX CORD secondary prevention study occurred between the end of 2012 and the beginning of 2014 [11].

Informed consents were obtained from healthcare personnel before survey administration. The Institutional Review Boards of University of Texas Health Science Center at Houston (UTHealth) and Baylor College of Medicine approved all protocols and procedures for the study (HSC-SPH-11–0513).

### Statistical analysis

All analyses were conducted by year of implementation (Year 1 or Year 2). We report frequencies with proportions to describe the characteristics of the providers. Mean scores with standard deviations for continuous variables and frequencies with proportions for categorical variables across the 42 survey items were computed. Mean scores were also reported for constructs with similar items.

#### Development of the implementation index

We computed an implementation index score from the 42 *Next Steps* items for each primary care clinic aggregated from the respective provider scores. A summative score was computed for items with similar constructs (four constructs: acceptability, adoption, appropriateness, and feasibility) at the provider level. Each of these summative scores was then converted to a percent score [percent score = (summative score)/(maximum potential score)]. For example, the summative score for feasibility construct was ‘x’ at a provider level. The maximum potential score for this construct was 4 (sum of the score of the 4 underlying binary items). The percent score for feasibility at the provider level would be [(100*x)/4]. The provider construct percent scores were then aggregated to the clinic level. We computed the implementation index score at the clinic level by taking the average of the construct percent scores. Only the surveys with ≥80% reported items were included in the score calculation (*n* = 5 surveys excluded). Cronbach’s alpha for the survey scales was calculated at both years.

#### Examination of implementation index score

We examined the mean implementation index scores with standard deviation (SD) for each year of implementation (Years 1 or 2). Mann-Whitney U-test was conducted to compare the mean implementation index score between the two years due to the small sample size.

#### Examination of association of implementation index levels with the enrollment of children with overweight and obesity in the TX CORD secondary prevention study

The 1957 referred patients from 11 primary care clinics were categorized as enrolled or not enrolled in the TX CORD secondary prevention study. The implementation index scores for the primary care clinics were converted into categorical variables with quartile splits collapsed into three categories: (a) relatively high level of implementation (upper quartile of the index), (b) medium level of implementation (middle two quartiles), and (c) relatively low level of implementation (lower quartile of the index). We then conducted a mixed effects logistic regression test with a random intercept term for primary care clinics to examine the association between implementation index levels and enrollment of children with overweight and obesity into the TX CORD secondary prevention study, separately for each year. Similar regression models were also used to examine the associations of underlying constructs, including acceptability, adoption, appropriateness, and feasibility with the enrollment of children into the TX CORD secondary prevention study. Significance was set at *P* value< 0.05. STATA software version 14.0 (College Station, TX) was used for data analysis.

## Results

### Provider characteristics

Table 1 presents the description of the PCPs by Years 1 and 2 of TX CORD clinic implementation. A total of 30 PCPs in Year 1 of implementation and 34 PCPs in Year 2 of implementation completed the survey across the 11 participating primary care clinics.

**Table 1:** Sociodemographic characteristics of primary care providers by year of implementation in the TX CORD study, 2013–2014

	Year 1 of implementation (2013), 30 (%)	Year 2 of implementation (2014), 34 (%)	*P* value
Female	25 (83.3)	26 (76.5)	0.496
At least 40 years of age	16 (53.3)	18 (52.9)	0.983
Race/ethnicity			
Non-Hispanic white	12 (40.0)	9 (26.5)	0.418
Non-Hispanic black	5 (16.7)	6 (17.7)	
Hispanic	4 (13.3)	10 (29.4)	
Others	8 (26.7)	9 (26.5)	
At least 6 years of employment in the current position	17 (56.7)	19 (55.9)	0.950
At least 6 years of experience in the medical field	25 (83.3)	26 (76.5)	0.348
At least 6 years of experience working with pediatric patients after completing medical training	25 (83.3)	26 (76.5)	0.496
More than 10 hours of prior training on how to manage childhood overweight or obesity	14 (46.7)	16 (47.1)	0.560

*TX CORD* Texas Childhood Obesity Research Demonstration

Missing data for all variables of interest was around 10%, thus, not reported in the table

### *Next steps* process evaluation data

Table 2 presents the descriptive information for the individual *Next Steps* items, separately for Year 1 and Year 2 of implementation. Overall, the providers felt the materials were acceptable, appropriate and feasible to use in treating patients with childhood overweight and obesity. However, they reported low to moderate adoption of the *Next Steps* program materials.

**Table 2:** Descriptive statistics of individual items included in the primary care clinic implementation index by year of implementation in the TX CORD study, 2013–2014

	Year 1 of implementation (2013)	Year 2 of implementation (2014)
Acceptability, mean (SD)		
Ease of learning to use the *Next Steps* materials		
I learned to use it quickly	2.8 (1.0)	2.6 (1.2)
I easily remember how to use it	2.6 (1.0)	2.5 (1.2)
The training I received was adequate	2.8 (0.8)	2.6 (1.1)
Satisfaction with *Next Steps* materials		
With overweight or obese kids, I am likely to use *Next Steps* during well child visits	2.8 (1.2)	2.2 (1.3)
With overweight or obese kids, I am likely to use *Next Steps* during acute care visits	1.7 (1.1)	1.5 (1.1)
With overweight or obese kids, I am likely to use *Next Steps* during obesity-specific visits	3.2 (1.1)	2.8 (1.2)
I am likely to use *Next Steps* with non- overweight/obese kids	1.8 (0.9)	1.7 (1.0)
Patients/families refer to the *Next Steps* poster	2.0 (0.9)	1.7 (1.0)
I refer to the *Next Steps* poster when I talk with patients/families	2.2 (1.0)	1.8 (1.0)
Mean score (SD) of the acceptability items at clinic provider level	2.5 (0.6)	19.4 (7.9)
Adoption, mean (SD)		
Frequency of use of *Next Steps* materials for counseling with patients who are overweight or obese?	1.7 (1.4)	1.4 (1.4)
How often do you use each of the following with patients who are overweight or obese?		
*Next Steps* poster	1.4 (1.3)	0.9 (1.2)
*Next Steps* flip chart	1.7 (1.4)	1.2 (1.4)
*Next Steps* activity books for home	0.9 (1.1)	0.6 (1.2)
List of community resources that support a healthy lifestyle	1.8 (1.3)	1.9 (1.7)
Mean score (SD) of the adoption items at clinic provider level	1.5 (1.0)	1.2 (1.1)
Appropriateness, mean (SD)		
When I talk with patients and families about obesity, the *Next Steps*		
Materials overall helps me be more effective	2.8 (0.9)	2.8 (1.1)
Poster helps me be more effective	2.3 (0.9)	2.4 (1.0)
Flip chart helps me be more effective	2.7 (1.0)	2.6 (1.1)
Patient activity book helps me be more effective	2.2 (1.0)	2.2 (0.9)
Materials fits into my patient/office flow	2.3 (1.0)	2.2 (1.1)
Mean score (SD) of the appropriateness items at clinic provider level	2.4 (0.7)	2.4 (0.9)
Appropriateness, mean (SD)		
Applicability of the themed visits, n (%)		
Category A: Setting the foundation		
Understanding health (set a foundation for good health) was applicable	17 (56.7)	22 (64.7)
Category B: Introduce concepts that are important to cover early for success		
Understanding healthy food was applicable	20 (66.7)	24 (70.6)
Home environment was applicable	17 (56.7)	20 (58.8)
Eating and your emotions was applicable	16 (53.3)	23 (67.7)
Portion sizes was applicable	20 (66.7)	25 (73.5)
Healthy drinks was applicable	21 (70.0)	24 (70.6)
Category C: Focus on other important concepts		
Physical activity was applicable	18 (60.0)	23 (67.7)
Feeling good about yourself was applicable	15 (50.0)	20 (58.8)
Reading food labels was applicable	16 (53.3)	21 (61.8)
Screen time and sleep was applicable	20 (66.7)	24 (70.6)
Meal patterns and snacks was applicable	18 (60.0)	24 (70.6)
Holidays/Special occasions was applicable	14 (46.7)	23 (67.7)
Eating away from home was applicable	17 (56.7)	22 (64.7)
Parenting was applicable	17 (56.7)	18 (52.9)
Community partners was applicable	10 (33.3)	21 (61.8)
Healthy family was applicable	17 (56.7)	22 (64.7)
Bullying and teasing was applicable	14 (46.7)	19 (55.9)
Goal setting was applicable	18 (60.0)	21 (61.8)
Unintentional disruptions was applicable	10 (33.3)	17 (50.0)
Mean score (SD) of the appropriateness items at clinic provider level	0.7 (0.4)	0.7 (0.4)
Feasibility, mean (SD)		
Access to the following materials provided by the CORD team, n (%)		
*Next Steps* poster was accessible	25 (83.3)	26 (76.5)
*Next Steps* flip chart was accessible	25 (83.3)	28 (82.4)
*Next Steps* activity books for home was accessible	23 (76.7)	20 (58.8)
List of community resources that support a healthy lifestyle was accessible	24 (80.0)	24 (70.6)
Mean score (SD) of the feasibility items at clinic provider level	0.9 (0.3)	0.7 (0.3)

*SD* Standard deviation, *TX CORD* Texas Childhood Obesity Research Demonstration

Acceptability scale: Strongly disagree (0), disagree (1), neither agree nor disagree (2), agree (3), and strongly agree (4)

Adoption scale: Less than 10% (0), 10–25% (1), 25–50% (2), 50–75% (3), and 75–100% (4)

Appropriateness scale measuring the effectiveness of *Next Steps* materials: Strongly disagree (0), disagree (1), neither agree nor disagree (2), agree (3), and strongly agree (4)

Appropriateness scale measuring the acceptability of themed visits: Keep as is/Rest of the groups

Feasibility Scale: Yes/No

### Implementation index score

Based on provider report, the implementation score for the *Next Steps* material was 63.2 (12.2)% at Year 1 and 55.3 (16.5)% at Year 2 across all clinics. The scores in Year 1 and Year 2 were not statistically different (*P* value = 0.375).

**Fig. 1:**
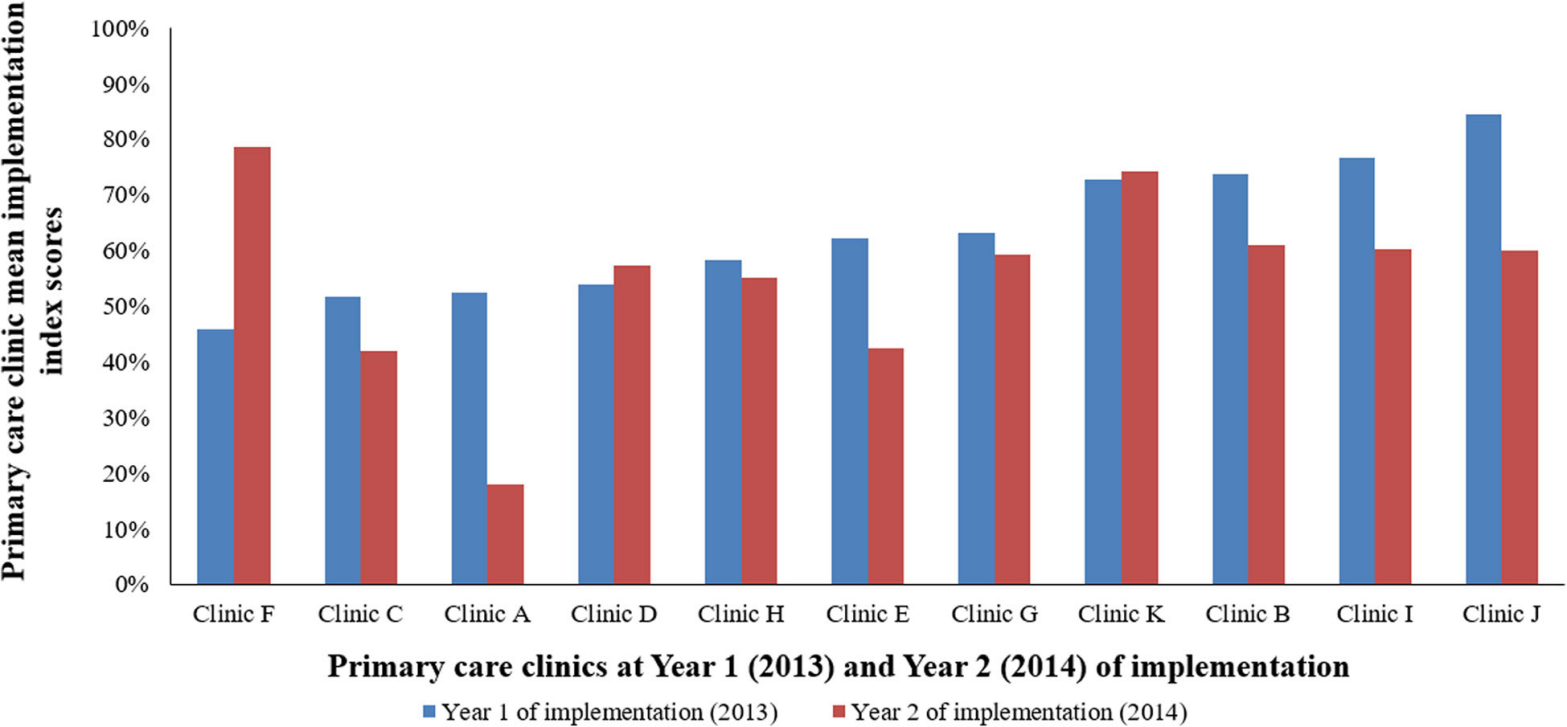
Primary care clinic implementation index scores (constructs included  acceptability, adoption, appropriateness, and feasibility), by year of implementation, TX CORD study

Figure 1 shows the range of implementation index scores across the primary care clinics, by years of implementation. The scores ranged from 45.9–84.4% in Year 1, compared to 18.0–78.5% in Year 2 of implementation. Cronbach’s alpha for the survey scales, including the individual constructs was high at both years (> 0.80).

### Implementation index levels and TX CORD secondary prevention enrollment

Implementation scores were categorized into three levels based on observed groupings at each year. Relatively low implementation was defined as scores in the lowest quartile (three clinics with score range in Year 1: 45.9–52.5% and Year 2: 18.0–42.4%). Relatively high implementation was defined as scores in the highest quartile (two clinics with score range in Year 1: 76.6–84.4% and Year 2: 74.3–78.5%).

Table 3 reports the frequency and proportions of children with overweight and obesity seen, referred, and enrolled from each clinic into the TX CORD secondary prevention study. Of the children with overweight and obesity seen at the 11 clinics (*n* = 6198), study referral rates by the individual clinics ranged from 8.5–66.8%. Of those referred (*n* = 1957), enrollment into TX CORD secondary prevention study from the individual clinics ranged from 18.3–50.0% [mean = 30.6 (11.7)%].

**Table 3:** Total number of children with overweight and obesity seen, referred, and enrolled into TX CORD secondary prevention study, 2013–2014

Clinics	Children with overweight and obesity seen (*n* = 6198)	Referred (*n* = 1957)	Enrolled (*n* = 533)	% referred (out of total seen)	% enrolled (out of total seen)	% enrolled (out of total referred)
Clinic I	1364	116	26	8.5%	1.9%	22.4%
Clinic C	436	82	15	18.8%	3.4%	18.3%
Clinic J	263	56	27	21.3%	10.3%	48.2%
Clinic D	677	171	61	25.3%	9.0%	35.7%
Clinic B	91	28	14	30.8%	15.4%	50.0%
Clinic F	540	194	34	35.9%	6.3%	17.5%
Clinic H	712	272	62	38.2%	8.7%	22.8%
Clinic K	716	293	77	40.9%	10.8%	26.3%
Clinic E	513	254	91	49.5%	17.7%	35.8%
Clinic G	684	356	74	52.0%	10.8%	20.8%
Clinic A	202	135	52	66.8%	25.7%	38.5%

*TX CORD* Texas Childhood Obesity Research Demonstration

Table 4 presents the results for the associations between clinic implementation index levels and enrollment of children with overweight and obesity into the TX CORD secondary prevention study. No significant relation was observed between implementation and enrollment into TX CORD secondary prevention study. Associations between underlying constructs of the implementation index and enrollment are also presented in Table 4.

**Table 4:** Odds of enrollment of children with overweight and obesity into TX CORD secondary prevention study by primary care clinic implementation index levels and by underlying constructs, 2013–2014

	Enrollment Year 1 (2013)	Enrollment Year 2 (2014)
	OR (95% CI)	OR (95% CI)
Implementation index (reference: relatively low implementation)		
Medium implementation	1.32 (0.71–2.47)	0.96 (0.53–1.76)
Relatively high implementation	1.62 (0.71–3.72)	0.61 (0.29–1.29)
Underlying constructs		
Acceptability	1.04 (1.00–1.08)	1.00 (0.98–1.02)
Adoption	1.01 (1.00–1.02)	0.99 (0.97–1.00)
Appropriateness	1.01 (0.99–1.03)	1.00 (0.98–1.01)
Feasibility	1.00 (0.99–1.02)	0.99 (0.98–0.99)

*CI* Confidence interval, *OR* Odds ratio, *TX CORD* Texas Childhood Obesity Research Demonstration

## Discussion

Our study examined the acceptability, adoption, appropriateness, and feasibility of the *Next Steps* program materials, which were pilot tested in primary care clinics that participated in the primary prevention component of the TX CORD study over two years of implementation. Implementation scores were lower in Year 2 than Year 1, though not statistically significant. There were no significant associations between levels of implementation of *Next Steps* and enrollment into TX CORD secondary prevention study.

We used a novel methodology to develop an index using process evaluation measures that assessed the implementation of weight management programs to improve the health of children with overweight and obesity in primary care clinics. Indices to assess implementation of child weight management programs in healthcare clinical settings are lacking; thus, our study is the first to develop such an index. Multiple studies have assessed the different components of a weight management program implementation such as reach, effectiveness, adoption, climate, cost, etc., in different settings [11, 19–21], although none has comprehensively assessed the implementation of the program with a single index score. The clinic index we developed was reliable (Cronbach’s alpha> 0.80) in assessing the underlying constructs; however, it warrants further psychometric analysis to include both measures of reliability and validity.

Although the providers in the current study perceived the *Next Steps* materials to be acceptable, appropriate and feasible, the frequency of use was around 10–50% of the time. The relatively low adoption of *Next Steps* material in the current study indicates that other factors led to a lower utilization of the materials during a clinic visit, such as limited time during a clinic visit, lack of training and retraining of providers, parental disengagement, providers’ preference for other obesity prevention approaches, lack of clinic administrative support for this protocol, or something as simple as materials being misplaced [11]. Future studies can assess the reasons for low adoption and then examine ways to increase providers’ use and accessibility to these materials [22].

The results of our study demonstrated some degree of fidelity of the *Next Steps* program materials across the primary care clinics, particularly during Year 1. Given that primary care clinics in this study primarily serve low-income populations, and since many factors influence these populations and their access to healthcare, implementing the *Next Steps* program 60% of the time in eight out of 11 clinics indicates the initial feasibility of the program in disadvantaged settings. Implementation scores were lower in Year 2 than Year 1, though not statistically significant. The providers received formal training in the *Next Steps* materials in 2012 when the primary care clinics were first recruited into TX CORD primary prevention study. During the Year 1 assessment of implementation, the research team was actively encouraging the providers to use the *Next Steps* tools with any potential patients that might benefit from them, as well as the recruiting tools for the TX CORD secondary prevention study. The second process evaluation data collection occurred 9–10 months after participant recruitment into TX CORD secondary prevention study had ended, when the research team was less visible in the clinics. Furthermore, 13.5% of the providers were in the primary care clinics for < 1 year in Year 2, and the new providers in Year 2 may not have been familiar with the material and study. These, along with the cross-sectional nature of the study and the perception of the TX CORD project as a study and not necessarily a program, might explain the decrease in the implementation of *Next Steps* materials between Year 1 and Year 2, and the inverse relationship observed between feasibility and enrollment in Year 2. Our results suggest the need for ways to increase engagement of providers such as repeat training of providers to increase the sustainability of *Next Steps* in primary care clinics [22].

There were no significant associations between levels of implementation of *Next Steps* and enrollment into TX CORD secondary prevention study. The potential effect of implementation of weight management materials on referral and enrollment of children into weight management programs is uncertain from this study as some of the largest clinics had a high implementation but low referral and enrollment, while the reverse was true in the small clinics (Table 3). The small number of clinics in this study limits the generalizability of the study, and the wide variation in percent of patients referred and enrolled in the TX CORD secondary prevention study, even within implementation level categories, delineates the need for future studies to understand the barriers and enablers to referral and enrollment to a weight management program both from provider and patient perspective [23]. For example, studies [24, 25] have reported that some of the provider-level barriers were lack of actual or perceived skill to discuss weight issues with patients and families and being ill-informed about existing services. At the patient-level [25–27], barriers included parents not perceiving the need for such programs for their children, parents not initiating the provider’s recommended care, lack of motivation from both parent and children, personal health problems, and logistics (e.g., scheduling issues, weather, transportation). Furthermore, referral to the program may be an inadequate outcome measure of implementation; the PCPs may have been using the weight management materials without referral of the children to the program. A more appropriate indicator of the use of *Next Steps* could be an improvement in BMI rather than enrollment into TX CORD secondary prevention. Because the TX CORD secondary prevention study was an experimental study that compared two weight management programs where *Next Steps* was the comparison group, we limited our outcome to an immediate one, enrollment of children with overweight and obesity into a weight management program.

There were certain limitations to our study. The study design was serial cross-sectional, thus, the implementation index scores at Year 1 came from a somewhat different cohort than in Year 2. Surveys with < 80% completion were excluded from the analysis (*n* = 5). This might introduce selection bias to the study findings if the non-responses were due to non-adherence with the *Next Steps* program. The process measures were self-reported, thus subject to recall or social desirability bias. Additional objective measures such as direct observations may provide a deeper insight into implementation fidelity of programs, and comparison of implementation of programs from different perspectives. The process measures were reported by the PCPs, so future studies could also assess the implementation of these measures by other clinicians such as dietitians or health educators. Only 11 primary care clinics participated in the study, providing limited extrapolation of study findings to other weight management primary care clinics. The TX CORD study was the first to pilot test *Next Steps* as a weight management program in primary care clinics, and there were no comparison clinics. Future studies should evaluate and validate the implementation of the *Next Steps* program in healthcare settings in a more rigorous design.

Despite these limitations, our study had strengths. This is the first study to develop a simple methodology for assessing implementation of weight management programs in healthcare settings. Survey scales had good internal consistencies at both years of implementation. Additionally, the implementation levels at the different primary care clinics demonstrated face validity. The *Next Steps* program materials were available in Spanish, thus, tailored towards the target population (predominantly, Hispanic/Latino). Finally, the homogeneity of the targeted population (2–12-year-old children from low-income underserved areas in Houston and Austin, Texas) also reflected a fair comparison of implementation of *Next Steps* program materials across the different primary care clinics.

## Conclusions

The results of our study highlight the importance of process evaluation and sustainability in primary care settings that are part of intervention programs. The implementation index scores created in this study provide an opportunity to assess how effective and comfortable the providers are at using the different weight-management tools in such settings. Using a standardized scoring procedure, we observed there was wide variability in the implementation levels across the primary care clinics that help to identify the best practices, barriers, and the need for modification of weight management tools tailored towards specific primary care clinics.

In conclusion, our study demonstrated an application of a novel implementation assessment technique to weight-management materials in primary care clinics in the TX CORD study.

## Abbreviations

AAP: American Academy of Pediatrics
BMI: Body Mass Index
CHIP: Children’s Health Insurance Program
EHR: Electronic Health Record
PCP: Primary Care Providers
SD: Standard Deviation
TX CORD: Texas Childhood Research Demonstration
UTHealth: University of Texas Health Science Center at Houston

## References

[1] Barriuso L, Miqueleiz E, Albaladejo R, Villanueva R, Santos JM, Regidor E (2015). Socioeconomic position and childhood-adolescent weight status in rich countries: a systematic review, 1990–2013. BMC Pediatr.

[2] Ogden CL, Carroll MD, Lawman HG (2016). Trends in obesity prevalence among children and adolescents in the United States, 1988-1994 through 2013-2014. JAMA.

[3] Bass R, Eneli I (2015). Severe childhood obesity: an under-recognised and growing health problem. Postgrad Med J.

[4] Finkelstein EA, Khavjou OA, Thompson H (2012). Obesity and severe obesity forecasts through 2030. Am J Prev Med.

[5] Huang TTK, Borowski LA, Liu B (2011). Pediatricians' and family Physicians' weight-related Care of Children in the U.S. Am J Prev Med.

[6] Barlow SE, Bobra SR, Elliott MB, Brownson RC, Haire-Joshu D (2007). Recognition of childhood overweight during health supervision visits: does BMI help pediatricians?. Obesity.

[7] Rabin BA, Brownson RC, Haire-Joshu D, Kreuter MW, Weaver NL (2008). A glossary for dissemination and implementation research in health. Journal of public health management and practice : JPHMP.

[8] Oude Luttikhuis H, Baur L, Jansen H (2009). Cochrane review: interventions for treating obesity in children. Evidence-Based Child Health: A Cochrane Review Journal.

[9] Profit J, Typpo KV, Hysong SJ, Woodard LD, Kallen MA, Petersen LA (2010). Improving benchmarking by using an explicit framework for the development of composite indicators: an example using pediatric quality of care. Implement Sci.

[10] National Initiative for Children's Health Care Quality. Next Steps: A Practitioner's Guide For Themed Follow-up Visits For Their Patients to Achieve a Healthy Weight. American Academy of Pediatrics Elk Grove Village, IL. 2013. https://shop.aap.org/next-steps-a-practitioners-guide-for-themed-follow-up-visits-for-their-patients-to-achieve-a-heal/. Accessed March 8 2017.

[11] Barlow SE, Butte NF, Hoelscher DM, Salahuddin M, Pont SJ (2017). Strategies to recruit a diverse low-income population to child weight management programs from primary care practices. Prev Chronic Dis.

[12] Hoelscher DM, Butte NF, Barlow SE (2015). Incorporating primary and secondary prevention approaches to address childhood obesity prevention and treatment in a low-income, ethnically diverse population: study design and demographic data from the Texas childhood obesity research demonstration (TX CORD) study. Childhood Obesity..

[13] Oluyomi AO, Byars A, Byrd-Williams C (2015). The utility of geographical information systems (GIS) in systems-oriented obesity intervention projects: the selection of comparable study sites for a quasi-experimental intervention design—TX CORD. Childhood Obesity.

[14] Taveras EM, Marshall R, Horan CM (2014). Improving children's obesity-related health care quality: process outcomes of a cluster-randomized controlled trial. Obesity (Silver Spring).

[15] Proctor E, Silmere H, Raghavan R (2011). Outcomes for implementation research: conceptual distinctions, measurement challenges, and research agenda. Adm Policy Ment Health Ment Health Serv Res.

[16] Rogers EM (1995). Diffusion of innovations.

[17] Glasgow RE (2003). Translating Research to Practice. Lessons learned, areas for improvement, and future directions.

[18] Glasgow RE, Vogt TM, Boles SM (1999). Evaluating the public health impact of health promotion interventions: the RE-AIM framework. Am J Public Health.

[19] Estabrooks PA, Wilson KE, McGuire TJ (2017). A quasi-experiment to assess the impact of a scalable, community-based weight loss program: combining reach, effectiveness, and cost. J Gen Intern Med.

[20] Johnson SE (2016). Pragmatic implementation trials: understanding the integrated research-practice partnership approach to lifestyle obesity management across a transforming health system: Virginia Tech.

[21] Parcel GS, Perry CL, Kelder SH (2003). School climate and the institutionalization of the CATCH program. Health Educ Behav.

[22] Chambers D, Simpson L, Neta G (2017). Proceedings from the 9th annual conference on the science of dissemination and implementation. Implement Sci.

[23] Perez AJ, Ball GDC. Helping children and families to enrol in weight management: what can stakeholders do? Paediatr Child Health 2018:pxy056-pxy. doi:10.1093/pch/pxy056 10.1093/pch/pxy056 PMC6376343 30792594

[24] Perez AJ, Kebbe M, Holt NL (2018). Parent recommendations to enhance enrollment in multidisciplinary clinical Care for Pediatric Weight Management. J Pediatr.

[25] Perez A, Holt N, Gokiert R (2015). Why don't families initiate treatment? A qualitative multicentre study investigating parents' reasons for declining paediatric weight management. Paediatr Child Health.

[26] Barlow SE, Ohlemeyer CL (2006). Parent reasons for nonreturn to a pediatric weight management program. Clin Pediatr.

[27] Perez AJ, Avis JL, Holt NL (2016). Why do families enrol in paediatric weight management? A parental perspective of reasons and facilitators. Child Care Health Dev.

